# The Reversal of Immune Exclusion Mediated by Tadalafil and an Anti-tumor Vaccine Also Induces PDL1 Upregulation in Recurrent Head and Neck Squamous Cell Carcinoma: Interim Analysis of a Phase I Clinical Trial

**DOI:** 10.3389/fimmu.2019.01206

**Published:** 2019-05-31

**Authors:** Donald T. Weed, Serena Zilio, Isildinha M. Reis, Zoukaa Sargi, Marianne Abouyared, Carmen R. Gomez-Fernandez, Francisco J. Civantos, Carla P. Rodriguez, Paolo Serafini

**Affiliations:** ^1^Department of Otolaryngology, Miller School of Medicine, University of Miami, Miami, FL, United States; ^2^Department of Microbiology and Immunology, Miller School of Medicine, University of Miami, Miami, FL, United States; ^3^Department of Public Health Sciences and Sylvester Biostatistics and Bioinformatics Core Resource, Miller School of Medicine, University of Miami, Miami, FL, United States; ^4^Department of Pathology and Laboratory Medicine, Miller School of Medicine, University of Miami, Miami, FL, United States

**Keywords:** myeloid derived suppressor cells, tadalafil, PDE5, mucin 1 vaccine, poly-ICLC, recurrent HNSCC, PDL1, immune exclusion

## Abstract

Myeloid Derived suppressor cells (MDSCs) play a key role in the progression and recurrence of human malignancies and in restraining the efficacy of adjuvant therapies. We have previously shown that Tadalafil lowers MDSCs and regulatory T cells (Treg) in the blood and in the tumor, primes a tumor specific immune response, and increases the number of activated intratumoral CD8^+^T cells in patients with primary Head and Neck Squamous Cell Carcinoma (HNSCC). However, despite these important immune modulatory actions, to date no clinically significant effects have been reported following PDE5 inhibition. Here we report for the first time interim results of our ongoing phase I clinical trial (NCT02544880) in patients with recurrent HNSCC to evaluate the safety of and immunological effects of combining Tadalafil with the antitumor vaccine composed of Mucin1 (MUC1) and polyICLC. The combined treatment of Tadalafil and MUC1/polyICLC vaccine was well-tolerated with no serious adverse events or treatment limiting toxicities. Immunologically, this trial also confirms the positive immunomodulation of Tadalafil in patients with recurrent HNSCC and suggests an adjuvant effect of the anti-tumor vaccine MUC1/polyICLC. Additionally, image cytometry analysis of scanned tumors indicates that the PDE5 inhibitor Tadalafil in conjunction with the MUC1/polyICLC vaccine effectively reduces the number of PDL1^+^macrophages present at the tumor edge, and increases the number of activated tumor infiltrating T cells, suggesting reversion of immune exclusion. However, this analysis shows also that CD163 negative cells within the tumor upregulate PDL1 after treatment, suggesting the instauration of additional mechanisms of immune evasion. In summary, our data confirm the safety and immunologic potential of PDE5 inhibition in HNSCC but also point to PDL1 as additional mechanism of tumor evasion. This supports the rationale for combining checkpoint and PDE5 inhibitors for the treatment of human malignancies.

## Introduction

The incidence of head and neck squamous cell carcinoma (HNSCC) has declined in the last 30 years but this remains a deadly disease with more than 550,000 cases and 380,000 deaths reported annually worldwide (1). Despite advances in diagnostic imaging, surgical ablative, and complex reconstructive techniques, radiation therapy, and chemotherapy, recurrence remains high and outcomes often poor for advanced stage disease. Treatment of recurrent HNSCC is challenging because it is constrained by previous therapies that debilitate the patient and greatly modify the treatment field. Additional treatments for salvage impose significant morbidities with potentially little, or even detrimental, impact on outcome (2). The lower probability of long-term cancer control, combined with higher toxicity of current treatment modalities in this setting (advanced stage recurrence at a fully treated site), often makes cure a less central, or even unachievable, goal of patient care. For patients with resectable recurrent tumor, surgical salvage remains the first-line and often only available treatment in previously irradiated patients. Although the addition of chemoradiotherapy as re-irradiation to salvage surgery improved locoregional control and disease-free survival, no differences were observed in overall survival because of more treatment-related deaths, distant metastases, and second primary tumors among the re-irradiated patients (3).

The absence or the high morbidity of effective adjuvant treatments in patients with advanced recurrent HNSCC undergoing salvage surgery is the primary reason for the poor prognosis of this disease (44% 2 year recurrence-free survival all stages) (4), and clearly indicates the need for new treatments characterized by low morbidity profiles and improved efficacy. Cancer immunotherapy has become widespread in recent years and is often used as first line of treatment in both solid and hematological malignances (5). Immune checkpoint inhibitors, in particular, have demonstrated considerable promise for the treatment of melanoma, non-small cell lung cancer, and other cancers (6). In recurrent HNSCC, only immune checkpoint inhibitors have proven clinical efficacy in randomized phase III trials with Nivolumab (anti-PD1) being the only immunotherapeutic drug approved for platinum-refractory recurrent/metastatic HNSCC (7). Despite this promising development, however, response rates of recurrent/metastatic HNSCC to Pembrolizumab (anti-PDL1) or Nivolumab are low (16 and 16.9%, respectively) (8, 9). The use of checkpoint inhibitors for recurrent HNSCC undergoing salvage surgery is currently being evaluated to determine whether immune modulation before and after surgery can eliminate minimal residual disease and prevent tumor recurrence (10, 11).

Immune exclusion (also known as the absence of effector T cells inside the neoplastic lesion) is emerging as one of the main reasons that may explain the lack of response in patients undergoing immunotherapies such as checkpoint inhibition therapy (12, 13). Immune exclusion seems to be particularly important for patients with T3/T4 tumors undergoing salvage surgery because of the absence of tumor infiltrating CD8^+^ T cells in ~60% of the patients and the absence of PDL1 expression in more than 90% of the tumors (14). Although the lack of immunogenicity of the tumor may play a role, the polarization and phenotype of myeloid cells infiltrating the tumor and in circulation seems to be a major determinant. Indeed, an elevated ratio between both monocytic or granulocytic myeloid derived suppressor cells (MDSC) and lymphocytes in the periphery and at the tumor site is also emerging as an important predictive factor in the response to checkpoint inhibitors across different types of malignancies such as melanoma, HNSCC, and non-small-cell lung and genitourinary cancers (15–22). Considering the fact that MDSCs promote tumor growth not only by providing immune protection to the tumor but also by regulating tumor angiogenesis and metastasis (23, 24), safe therapeutic strategies aimed to inactivate, deplete, or convert these cells are highly desirable to further build on the success of checkpoint inhibitors and extend the number of patients that may benefit from immune therapeutic interventions. Indeed, in preclinical models, their functional inhibition is sufficient to restore the efficacy of anti-PDL1 antibodies (25). Furthermore, MDSCs and macrophages infiltrating the tumor express PDL1 and often are the major population in the tumor expressing this ligand (15). Thus, it is possible that strategies designed to eliminate/inhibit MDSCs and macrophages may even be sufficient to reverse T cell exhaustion and promote tumor rejection.

PDE5 inhibition, via repurposing of drugs commonly used for the treatment of erectile dysfunction, is an emerging experimental option that has been and is being tested in different clinical trials to lower MDSCs and prime or unleash the spontaneous anti-tumor immune response. In our original preclinical works, we showed that the PDE5 inhibitor sildenafil effectively inhibits MDSCs by increasing cGMP and reducing their expression of arginase 1, Nitric oxide synthase 2, and IL4Rα in mouse models of mammary carcinoma, colon cancer, and fibrosarcoma (26). PDE5 inhibition was sufficient to prime a spontaneous anti-tumor response, increase the number of tumor infiltrating T cells, and significantly decrease tumor progression (26). Furthermore, in a lymphoma model, we demonstrated that tumor progression and the accumulation of tumor specific Treg accumulation correlated with the expression of IL4Rα in MDSCs (27). In this model, sildenafil, by lowering MDSCs activity, was sufficient to inhibit IL4Rα expression on MDSCs, reverse T cell anergy, and reduce the number of tumor specific Tregs (27). These data were then independently confirmed by different groups in colon carcinoma, spontaneous prostate cancer, melanoma, and metastatic mammary adenocarcinoma models (28–31).

In our previous double blinded, randomized, placebo controlled, phase1/2, independent clinical trials in HNSCC (NCT00894413, NCT00843635) (32, 33), Tadalafil was given daily pre-operatively for 14 (10 mg/day NCT00894413) or 21 (10 or 20 mg, NCT00843635) days. In both clinical trials, Tadalafil treatment was well-tolerated, with back pain and painful myalgias (all symptoms resolved within 48 h after treatment discontinuation) as a major side effect in a small percentage of subjects receiving the study drug. Analysis of cryopreserved PBMCs showed a significant reduction in both monocytic MDSCs and Treg (Supplementary Figures 1A,B) confirming in humans (32, 33) the immunomodulatory activity of Tadalafil observed in preclinical models (26, 27). Treatment was also associated with the reversal of systemic immunosuppression shown by a significant increase of the DTH response to recall antigens and upregulation of ζ-chain on CD8^+^T cells (32). Furthermore, chronic PDE5 inhibition, significantly increased the anti-tumor T cell response evaluated by assessing the proliferation of magnetically purified CD3 T cells isolated before and after treatment to autologous dendritic cells pulsed with the autologous tumor (33). At the tumor site, treatment decreased MDSCs and Treg, and increased the number of CD69^+^CTL and effector CD4^+^ cells (33). Interestingly, further data analysis suggested that these positive effects were maximized at intermediate drug dosage (range 145–225 μg/Kg) possibly because of an off-target effect of Tadalafil on PDE11 at higher dose (33). However, despite these positive immunological effects and the surgical resection of the tumors, Tadalafil as monotherapy in a neoadjuvant setting, did not dramatically increase recurrence free survival in the treated patients as revealed by our analysis of the NCT00843635 trial (Supplementary Figure 1C).

Taken together these studies indicate that PDE5 inhibition positively modulates tumor immunity by reducing the systemic immunosuppression, by priming an anti-tumor immune response, and by increasing the infiltration of effector T cells in the tumor. However, to date, these studies have failed to demonstrate a dramatic clinical benefit of Tadalafil treatment in cancer patients.

Here we evaluate whether the combination of Tadalafil and an anti-mucin (MUC) 1 vaccine with poly ICLC as adjuvant is safe and can reverse immune exclusion in patients with recurrent stage 3 and 4 HNSCC as an interim analysis of a phase I clinical trial (NCT02544880). This trial is designed as a phase I lead-in in anticipation of a randomized phase II trial to compare the combination of Tadalafil and the anti MUC1/polyICLC vaccine with each therapy individually and in comparison with a non-randomized control group of patients undergoing surgical salvage alone.

MUC1 has been identified by the NCI as one of the top promising targets for cancer vaccines(34), as it is present in most of T2-T3 HNSCCs, and its expression is associated with tumor aggressiveness, lymph node metastases and a poor prognosis (35–41). While in normal tissues MUC1 is fully glycosylated and thus it is invisible to the immune system, in HNSCC this transmembrane protein is overexpressed and under-glycosylated (35–40). Importantly, MUC1 has been identified by a bead-based affinity-fractionated proteomic method as the immune dominant antigen for CD4 and CD8 T cells in 80% of patients with HNSCC (42). Clinical trials performed with MUC1 vaccines in patients with cancer showed an excellent safety profile with no sign of autoimmunity or serious side effects and encouraging results for less immunosuppressed patients (43–47). However, lack of response to the vaccine was observed in patients without cancer but with a history of premalignant lesions such as advanced colon adenomas and was further characterized by an elevated concentration of MDSCs in the blood of non-responding patients (47), suggesting a rationale for simultaneous PDE5 inhibition (to lower MDSCs) and anti-MUC1 vaccination.

## Materials and Methods

### Clinical Trial Schema and Patient Enrollment

A phase I clinical trial (NCT02544880) is being conducted following the protocol approved by the IRB of the University of Miami and under the IND 16403. Patients undergoing salvage surgery with biopsy-proven, surgically resectable, recurrent or second primary HNSCC of the oral cavity, oropharynx, hypopharynx or larynx, recurrent stage III-IV, and whose recurrent tumors were within a previously irradiated field, were eligible for the trial. We excluded patients with distant metastatic disease, those that underwent prior immunotherapy with checkpoint inhibitors, those that used PDE5 inhibitors in the 2 weeks before enrollment, those with prior or known adverse reaction to PDE5 inhibitors, those immunocompromised for reasons not directly related to patient's malignancy, and those for which the study drugs are not recommended based on other clinical comorbidities. Additionally, to eliminate confounding variables, we excluded patients with hepatitis -B, -C or HIV, those with a history of severe autoimmune disease, female patients who were pregnant or breastfeeding, or patients in vulnerable subject categories. A complete list of inclusion and exclusion criteria is provided in Supplementary Table 1. This phase I trial was designed to accrue six evaluable patients as a lead-in for a three arm randomized phase II trial comparing the combination treatment of Tadalafil and the anti MUC1/poly ICLC vaccine with single modality treatment of either Tadalafil or the anti-MUC1/polyICLC vaccine, in addition to a fourth non-randomized control arm of otherwise eligible patients undergoing salvage surgery but unwilling to take study related drugs. The phase I lead-in and randomized phase II trials were designed to allow accrual to the non-randomized control arm to begin during the phase I lead-in should eligible patients for the control arm present during the enrollment period of the phase I in order to maximize accrual to the control arm for the phase II trial. Eligible patients were enrolled to the non-randomized active treatment arm or as non-randomized controls after signing the appropriate IRB approved informed consent.

**Figure 1:**
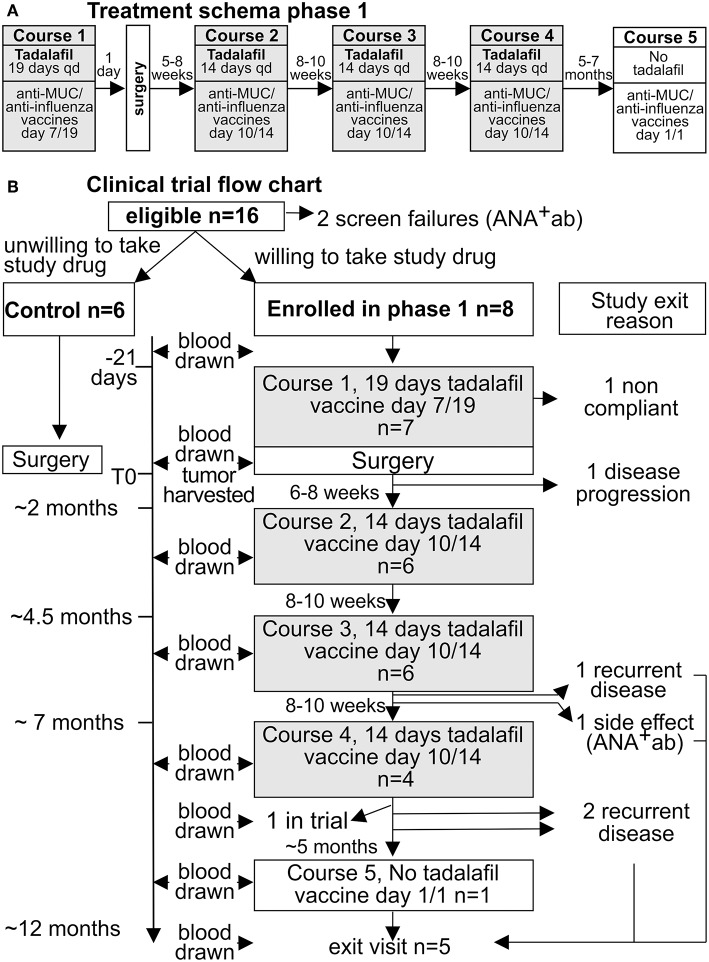
Flow diagram and design of the phase 1 study.

Following enrollment, patients in the experimental group were treated with Tadalafil for 19 days pre-operatively with anti-MUC1/poly ICLC vaccine given on day 7 of the Tadalafil course. Salvage surgery was performed 21 days following initiation of Tadalafil. Three additional courses of Tadalafil of 14 day durations with anti-MUC1/poly ICLC vaccine given on day 10 of each course were completed at ~2, 4, and 6 months post-operative. A final anti-MUC1/ poly ICLC vaccine was given at 1 year post-operative. All patients were to be followed for 1 year beyond the end of course 5 or until withdrawn from the study for recurrence, TLT, death, or other reason. The study schema and flow chart is diagramed Figure 1.

For those patients assigned to the experimental group, enrollment was designed in a sequential fashion based upon Treatment Limiting Toxicities (TLTs) occurring during the first 2 Courses of treatment, in a manner such that no more than 2 patients were allowed to have TLTs at the same time. At least one of the first 2 patients enrolled must have been evaluated for TLT(s) up to the end of their Course 2 treatment, before patient 3 could begin Course 1 treatment. Patient 3 was allowed to begin Course 1 treatment if the first patient to complete evaluation for TLT's at the end of their Course 2 treatment did not experience a TLT. If neither patient 1 nor patient 2 experienced a TLT up to the end of both of their completion of Course 2 then patient 4 was allowed to begin Course 1 treatment. These same conditions applied for patients 5 and 6 beginning their Course 1 treatment (if no TLT was noted through completion of Course 2 for patients 1 and 2, and for either patient 3 or 4, then patient 5 would be allowed to begin Course 1, if neither patients 3 nor 4 experienced TLT after completion of both of their Course 2 treatments then patient 6 may begin Course 1 treatment). On the other hand if any patient experienced a TLT through the end of Course 2 then all subsequent patients would begin Course 1 treatment only after all prior patients had completed Course 2 with no additional TLTs identified.

Study subjects were considered evaluable for phase I safety analysis once they completed Course 2 or if they experienced a TLT prior to completion of Course 2. The phase I lead in was designed to accrue 6 evaluable patients for safety analysis. This safety analysis was planned following completion of Course 2 for the sixth evaluable study subject. An interim analysis of preliminary immunologic endpoints of all subjects enrolled in the phase I trial inclusive of those non-randomized controls enrolled during this same time period was planned to coincide with the safety analysis. Results of these combined analyses (safety and immunologic endpoints) were to be utilized to inform a decision whether or not to proceed with accrual to the randomized phase II trial. These results are presented below.

### Monitoring for Adverse Events

The NCI common terminology criteria for adverse events (CTCAE3.0) were used to monitor toxicity. Laboratory monitoring, including CBC, BUN, creatinine, liver function tests, and ANA test were performed at baseline, before each treatment course and 14 days following course 4 and course 5, as well as 14 days following withdrawal from the study for other reasons such as recurrence or adverse event. Safety questionnaire was completed between 5 and 12 days following vaccination in each treatment course. A final safety questionnaire was administered at 24 months post-operative for those patients alive. Patients were questioned regarding adverse events with each follow up clinical evaluation for monitoring of their cancer status as per standard of care, including appropriate physical examination to assess disease status. Follow-up imaging was performed as clinically indicated. A treatment limiting toxicity (TLT) was defined as any one of the following adverse events (AEs) and was attributed (*possible, probable, or definite*) to the combination of Tadalafil/Vaccine treatment. Treatment discontinuation was required if a patient experienced a TLT. TLTs included new or worsening autoimmune disorder Grade ≥2, allergic reactions Grade ≥2 (Grade 2 drug fever considered an exception), and any other Grade ≥3 toxicity that in the opinion of the Investigator required discontinuation of study treatment. Exceptions included Grade ≥3 transient myalgia, back pain, or reversible hypotension, all of which were not considered a TLT if lasting <5 days. Patients were considered evaluable for safety who received at least one dose of Tadalafil, while patients were considered evaluable for TLTs who either experienced a TLT up to the end of Course 2 or received all scheduled doses of treatment through completion of Course 2 without TLT. The Sylvester Comprehensive Cancer Center (SCCC) Data and Safety Monitoring Committee (DSMC) monitored this clinical trial according to the Cancer Center's DSM Plan on a quarterly basis.

### Specimen Collection

Blood (~50 mL) was drawn in EDTA-containing tubes at baseline, at the day of surgery (after treatment); during tadalafil treatment on day 10/14 on course 2, 3, and 4; 15 days after treatment of course 4, and at course 5. Additional blood draws were performed at the exit visit either ~15 days after course 5 or when a subject was withdrawn from the study because of recurrence, adverse events, or other reason. All specimens were processed within 2 h of being harvested. Fresh tumor specimen (at least 14 mm^3^) was collected at the time of definitive tumor resection for tumor lysate preparation, and was processed within 1 h of harvesting. Additional specimens from available pretreatment biopsy and surgery were paraffin-embedded for immunofluorescence studies. For the control patients, blood was harvested before surgery and in eventual follow up visits.

### Tadalafil Treatment and Dose

Tadalafil (Cialis™, Eli Lilly) was purchased through the UM clinical pharmacy and given orally q.d. at a weight-normalized dose as follow: 10 mg/day if weight ≤63.5 kg, 15 mg/day if weight >63.5 kg and ≤104.3 kg), or 20 mg/day for weight >104.3 kg as suggested in Weed et al. (33).

### Vaccines and Immunization

Patients were immunized intramuscularly on day 7 of 20 in course 1, day 10 of 14 course 2–4, and day 1 of 1 in course 5 against MUC1 and, when seasonally available, influenza vaccine.

The MUC1 vaccine was composed of 50 μl of the MUC1 100 mer peptide (H2N-5X(GVTSAPDTRPAPGSTAPPAH-CONH2, [2 μg/μl], kindly provided as a gift by Dr. O. Finn, University of Pittsburg) admixed with 250 μl of POLY-ICLC (Hiltonol®, [2 μg/μl] provided by Oncovir at production cost) for total volume of 300 μl.

The influenza vaccine flublock, composed of recombinant proteins, was provided by Protein Sciences Corporation.

### Dendritic Cells Preparation

Monocytes from freshly drawn PBMCs were isolated by adherence in a T75 flask (BD) for 2 h in RPMI-1640 containing 1% heat-inactivated human AB serum. Following washing to remove non-adherent cells, the adherent monocytes were differentiated into DC with RPMI-1640 1% AB serum containing 800 U/mL GM-CSF and 500 U/mL IL4 (Peprotech) for 5 days. Fresh GM-CSF and IL4 was added on day 3. On day 5, immature DC were transferred into 24-well plates and pulsed with MUC1 peptide (10 μg/mL) in RPMI-1640 1%AB serum supplemented with GM-CSF and IL4. Two hours later, pulsed immature DC were induce to mature by the addition of Mimic cytokine mix [5 ng/mL TNFα (Peprotech), 5 ng/mL IL1β (Peprotech), 750 ng/mL IL6 (Peprotech), and 1 μg/mL PGE2 (Sigma)].

### Magnetic Sorting

CD3^+^ T cells were purified by negative selection using the human Pan T Cell Isolation Kit II (Miltenyi Biotec) in combination with the LS column and following the manufacturer's instruction. Purity was evaluated by FACS and was generally higher than 90%.

### Functional Assays

Magnetically purified, CFSE-labeled T cells (10^5^) from baseline (Course 1 before treatment), or from 15 days after Course 4 were incubated with 3 × 10^5^ autologous, monocyte-derived, DC pulsed with the MUC1 peptide. T-cell proliferation was evaluated by flow cytometry 4 days later.

### Flow Cytometry

Flow cytometry was performed on whole blood and freshly ficolled PBMCs of patients at each time point. Data acquisition was performed on aBD LSRII equipped with the following wavelengths lasers: 405 nm (50 mW), 488 nm (50 mW), 532 nm (150 mW), and 640 nm (40 mW). MDSC phenotype analysis was performed using Zombie Violet™ Fixable Viability Dye (BioLegend) and the following anti-human Abs: CD33-FITC (clone HIM3-4; BD), Lox1-APC (clone 15C4; BioLegend), CD124-PE (clone 25463; R&D Systems), CD14-APC-H7 (clone MϕP9; BD), CD15-BV711 (clone W6D3; BD), HLA-DR V500 (clone G46-6; BD), CD11b-BV605 (clone ICRF44; BD). T-cell analysis was performed using Zombie Violet™ Fixable Viability Dye (BioLegend) with the following antibodies: CD3-Alexa Fluor 700 (clone OKT3; eBioscience), CD247-PE (clone 6B10.2; eBioscience), CD4-BV711 (clone SK3; BD), CD8-BV605 (clone SK1; BD), CD69-APC-Cy7 (clone FN50; BD), Foxp3-APC (clone 236A/E7; e-Bioscience), CD154-PE/Dazzle 594 (clone24-31; BioLegend). For the staining, 150 μl of whole blood or 5 × 105 ficolled PBMCs at 4°C, were admixed with 123-counting beads (e-bioscience) and the optimized antibodies cocktail for 30' at 4°C. Cells were washed with PBSand, lysed with 2 ml of ACK (Gibco) at RT for 15', washed twice with PBS, and labeleled with LIVE/DEAD staining. For T cell staining were then fixed and permeabilized and stained for Foxp3 using the Foxp3/Transcription Factor Staining Buffer Set (e-Bioscience) and following the manufacturer's instructions. Samples were read in the cytofluorimeter within 2 h of staining. At least 105 events were collected. Compensation was performed using compi-beads (BD) after data collection. FMO were used as negative controls. Data were analyzed using the FCS vs6 (*denovo* software). Gating strategy are summarized in Supplementary Figure 3.

### MUC1 IHC

IHC was performed as described in Cascio et al. (48). Briefly, deparaffinized and rehydrated 4 μm sections of tumor specimen were incubated for 15 min at RT in a 30%H_2_O_2_/methanol solution (1:10) to block endogenous peroxidase activity. Slide were washed 3 times with PBS 1X, antigens were retrieved in 0.1% citrate buffer pH 6 for 5'at 120°C. Sections were permeabilized in PBS-0.2% Tween20 (5'at RT)and incubated with incubated PBS-2%BSA (20' at RT) to block non-specific binding. Samples were then incubated 1 h RT with a 1:40 dilution in PBS−2% BSA of the anti Mucin 1 antibody that specifically recognizes the underglycosylated, tumor specific form, of MUC1 (VU-4H5, Santa Cruz Biotechnology). Slides were washed in PBS-0.2% TWEEN20 (5'at RT) and incubated for 1 h with the biotinylated anti-mouse IgG secondary antibody (Vector Laboratories dilution 1:200 in PBS-BSA2%) and washed in PBS-0.2% TWEEN 20 for 5'at RT. Slices were incubated with ABC solution (Vector Laboratories) for 30'at RT washed, developed with DAB substrate (BD Pharmingen).

### Image Cytometry

Four μm sections of tumor specimen were deparaffinized, rehydrated, and incubated for 30 min at RT in a sodium borohydride solution (0.5 mg/mL in PBS; EMDGibbstown, NJ, USA) to reduce auto fluorescence. Antigen retrieval was performed by a 5 min incubation at 120°C in EDTA antigen retrieval solution pH = 9 (GIBCO Carlsbad, CA, USA). Slides were then incubated with Image-iT (Invitrogen) for 30 min at RT followed by incubation (1 h at RT) with PBS containing 1% BSA and 0.05% Triton-X100 to permeabilize the tissue and block non-specific binding. Samples were incubated O/N at 4°C with the primary antibodies diluted in PBS with 1% BSA. After three washes with PBS, samples were labeled for 2 h at RT with the relevant secondary antibodies, counterstained in PBS containing 2 mM DAPI (Invitrogen), for 30 min at RT, and rinsed with PBS. Coverslips were mounted using Biomeda gel mounting media (Electron Microscopy sciences, Hatfield, PA, USA). The following primary antibodies were used: mouse monoclonal anti-human FOXP3 antibody (clone 237/E7, dilution 1/25, Abcam) and the goat polyclonal anti-human CD4 antibody, (dilution 1/20, R&D System). Rabbit polyclonal anti-human CD33 antibody (dilution 1/15, Santa Cruz Biotechnology) and mouse monoclonal anti-human IL4Rα antibody (clone 25463, dilution 1/15, R&D System). Rabbit polyclonal anti-human CD8 antibody (dilution 1/30, Abcam) and goat polyclonal anti-human CD69 antibody (dilution 1/25, R&D System). Mouse monoclonal anti-human CD163 antibody (clone 10D6 dilution 1/100, Leica Biosystem) and rabbit monoclonal anti-human PD-L1 antibody (clone SP142, dilution 1/50, Abcam). As secondary antibodies we used: Alexa Fluor-555 conjugated anti-mouse antibody (for FoxP3, CD163 and IL4Ra, Invitrogen); Alexa Fluor-488 conjugated anti-goat antibody (for CD4 and CD69, Invitrogen); Alexa Fluor-555 conjugated anti-rabbit antibody (for CD8, Invitrogen); Alexa Fluor-488 conjugated anti-rabbit antibody (for CD33 and PD-L1, Invitrogen) all secondary antibodies were diluted 1/500 in PBS/BSA 1%. Stained slides were scanned at 20X with an Olympus VS120 microscope (Olympus) using a DAPI CUBE 455 nm, a FITC CUBE 518 nm and a TRITC CUBE 580 nm. Images for each patient were exported as single channel tiff files with OlyVIA software with a resolution of 5x and qualitatively evaluated with ImageJ (https://fiji.sc/) and processed with cell-profiler (www.cellprofiler.com) and fed into FCS Express 6 plus (https://www.denovosoftware.com/site/Plus-Overview.shtml). Detailed on image processing are provided in Supplementary Material section.

### ELISA

IgG, IgA, and IgM levels were examined in the plasma of patients for each time point as previously described (47). Briefly, Immulon plates were coated with 1 μg/well of MUC1 peptide or recombinant influenza proteins (Flublock) and incubated overnight at 4°C. The next day, plates were washed and then incubated at room temperature (RT) for 1 h with blocking buffer (DPBS-BSA 2.5%). Blocking buffer was discarded and 50 μl of plasma (diluted 1:40 in blocking buffer) was added to the plates (in duplicates) and incubated at RT for 1 h. After another washing step, 50 μl of diluted goat anti Human-HRP IgA; IgG or IGM secondary antibody (for MUC1) or a combination of the three antibodies (HA) was added to each well, and plates were incubated at RT for 1 h. Plates were washed and a 100 μl of substrate solution (SIGMA-FAST p-Nitrophenyl) was added to each well and plates were incubated for 20 min at RT followed by the addition of 50 μl of stop solution (NaOH 0.5 M). Absorbance was measured at 405 and 410 nm wavelength.

### Statistical Analysis

Statistical analysis was performed in coordination with the statistical core at the Sylvester cancer center. Time comparison within a treatment arm was assessed by paired *t*-test or RM-one way ANOVA. Comparisons between treatment arms were done by two-sample *t*-tests or ANOVA, or by non-parametric methods, the Mann–Whitney or Kruskal–Wallis test. All tests were two-sided with 5% significance statistical analysis. An interim safety analysis was planned after the 6th patient in phase I completed Course 2 (of planned 5 treatment courses). This interim analysis was planned to evaluate safety with attention to occurrence of TLTs, other AEs, as well as clinical data such as recurrence, and preliminary analysis of immunologic endpoints. The results of this interim analysis are summarized in this report. Recurrence-Free Survival (RFS) was evaluated for RFS by follow-up assessment(s) post-surgery as per routine care. RFS is defined as the time from date of Surgery to the date of first documented recurrence. Recurrence was demonstrated by clinical assessments such as clinical examinations and tumor assessments (possibly) by CT, PET/CT or MRI. Patients under follow-up and those lost to follow-up have been censored at the last date of documented recurrence-free status. Correlations were evaluated by Pearson correlation analysis. Statistical analyses were conducted using SAS software version 9.2 (SAS) or Sigmaplot vs12.5.

## Results

### Underglycosylated Muc1 Is a Tumor-Associated Antigen Widely Expressed in Patients With Recurrent HNSCC

**Figure 2:**
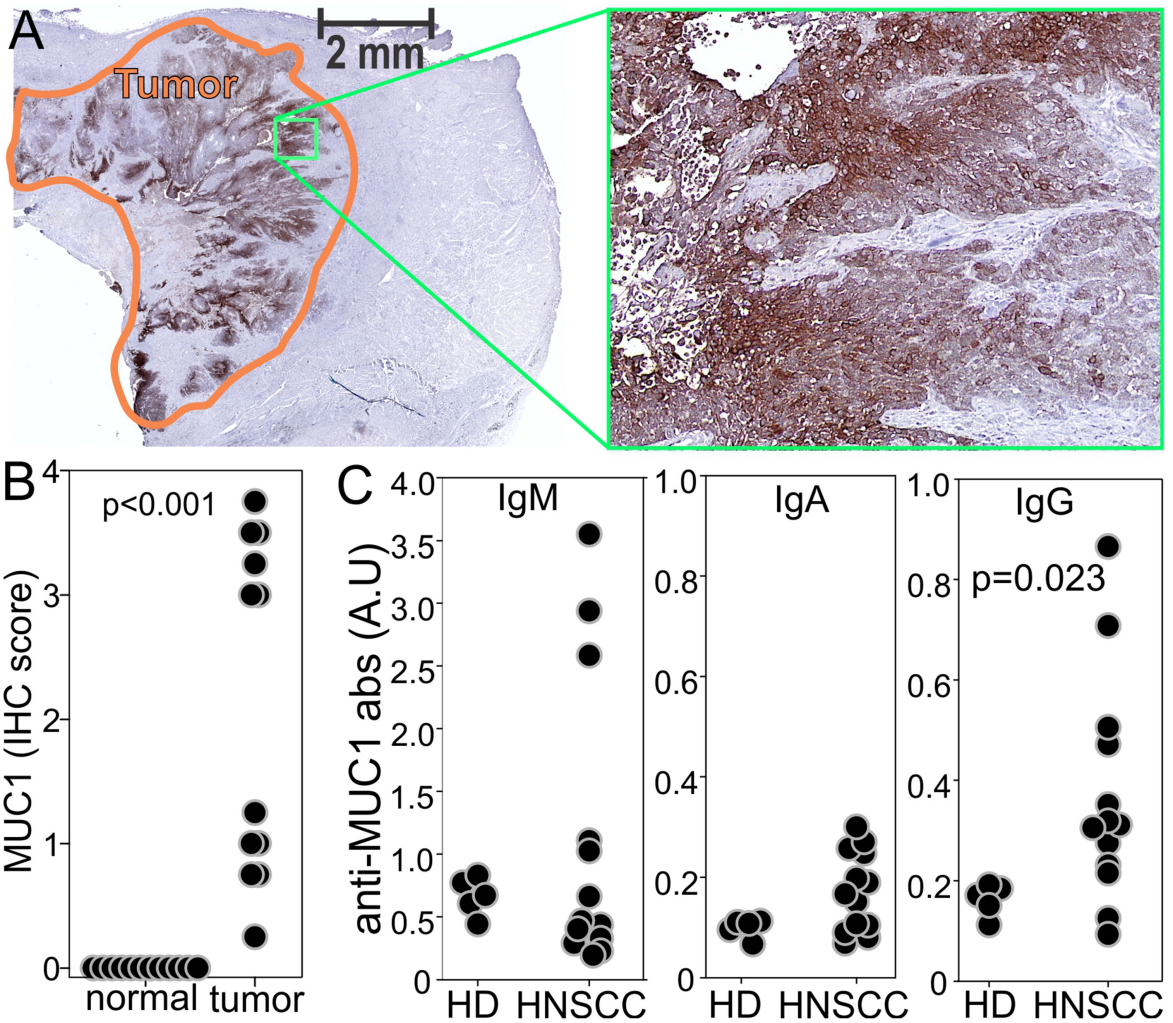
Underglycosylated MUC1 is a common tumor specific antigen in stage 3/4 recurrent HNSCC. **(A)** MUC1 was evaluated by IHC on slides from the tumor specimens of enrolled patients. **(B)** Tumors and surrounding “normal” tissues were scored from 0 to 4 independently by four investigators based on the staining intensity and extension, and individual scores averaged. Examples of different scores are reported in Supplementary Figure 2. **(C)** ELISA for MUC1 specific antibodies was performed on the sera of healthy donors or enrolled patients at baseline.

Underglycosylated MUC1 has been proposed as a tumor associated antigen in HNSCC, however, its expression in recurrent HNSCC has not been analyzed. Thus, we performed IHC on the tumor specimens and each staining was independently scored from 0 (no staining) to 4 (strong homogenous staining) by four experienced investigators. Scores were averaged, and examples of staining and relative scores are reported in Supplementary Figure 2. Underglycosylated MUC1 was found expressed in most of the analyzed specimens whereas it was undetectable in the “normal” tissue surrounding the neoplastic lesions (Figures 2A,B). This analysis supports the notion of underglycosylated MUC1 as a tumor specific antigen in patients with recurrent HNSCC. We next evaluated whether a preexisting immunity was present in these patients as well as in healthy donors. Anti-MUC1 IgG antibodies were significantly higher in the sera of recurrent HNSCC patients compared to one of age matched healthy controls (Figure 2C) suggesting the presence of a memory response against this antigen. Taken together, these data suggest that underglycosylated MUC1 is an immunogenic tumor specific antigen in recurrent HNSCC.

### Tadalafil and MUC1/polyICLC Vaccine Are Well-Tolerated in Patients With Recurrent HNSCC

Although MUC1 vaccine and PDE5 inhibitors has been proven safe when used as monotherapy in cancer patient the safety of the combination of these two immunologic strategies has not been previously evaluated. Thus, we performed a proof of principle, phase I clinical trial (NCT02544880) with safety and immunological endpoints in patients with recurrent stage 3 and 4 HNSCC undergoing salvage surgery (Figure 1). As controls we enrolled eligible patients willing to donate blood and tumor specimen but unwilling to receive study drugs. This cohort of patients was chosen because of the absence or the high morbidity of effective non-surgical treatments as alternatives to or adjuvant to standard of care salvage surgery, and the high recurrence rate (up to 70%) associated with salvage surgery alone in patients with recurrent, resectable, advanced staged HNSCC in a previously irradiated field (49). Even a small trial in this population might provide some insights regarding the clinical efficacy of an experimental treatment. Patient demographics, clinical characteristics, and complete list of inclusion and exclusion criteria are reported in Supplementary Tables 1, 2.

In this trial, patients received 4 courses of Tadalafil (orally q.d.) in association with MUC1/polyICLC vaccine (i.m. 1 week after each Tadalafil treatment initiation). The first course (19 days) was given in a neoadjuvant setting whereas course 2, 3, and 4 (14 days each) were given ~5, 12, and 21 weeks after salvage surgery. One year after surgery (course 5), patients received the MUC1/polyICLC vaccine without Tadalafil.

A total of 14 patients have been enrolled on this trial, 6 as control and 8 patients to the active treatment arm. Two patients on the active treatment arm were not evaluable for treatment limiting toxicity (TLT) analysis. Subjects were evaluable for TLT if they completed at least two courses of study drug or if they developed a TLT at any time prior to completion of course 2. One (subject 1–01) developed disease progression following early tumor recurrence after lengthy recovery from surgery and was withdrawn from the study without receiving course 2 study treatment. The other patient was non-compliant with study drug administration and was withdrawn at the beginning of course 1. Disease sites for 6 evaluable treatment patients included oral cavity (*n* = 2), oropharynx (*n* = 2), and larynx (*n* = 2), while disease sites of control patients included oral cavity (*n* = 4), oropharynx (*n* = 1), and larynx (*n* = 1). Recurrent tumor summary stage for active treatment patients were IV (*n* = 5) and III (*n* = 1), while all control patient recurrent summary stage was IV (*n* = 6). Three of the 12 enrolled patients had p16 positive tumors consistent with HPV related malignancies, including 1 control and 2 treatment patients. All patients were previously irradiated as per study protocol, with 4 of 6 on the active treatment arm and 5 of 6 on the control arm receiving chemotherapy with radiation therapy as part of their prior treatment.

A total of 27 grade 1 or 2 adverse events (AEs) were recorded in the 8 phase 1 patients who received any study drug (Supplementary Table 3) and included flushing (1), headache (3), myalgia (1), nausea (1), vomiting (1), and an asymptomatic autoimmune disorder (1) as revealed by the development of anti-nuclear antibodies (ANA) while on treatment. The subject was electively withdrawn from the active treatment arm of the trial, and subsequent ANA testing of this subject has reverted to normal. No TLTs were recorded in the 6 patients evaluable for TLT. One of 6 evaluable active treatment patients continues to receive treatment on trial, having just completed course 4 with no evidence of recurrence. One of the remaining 5 active treatment patients completed all treatment courses but developed recurrence in the second year of follow-up. One of the remaining 5 active treatment patients was withdrawn from the study due to the development of a positive ANA, with no evidence of recurrence. The remaining three active treatment patients developed recurrence prior to completion of all study courses. Thus, far overall recurrence free survival of treated and control patients is similar (Supplementary Figure 6) but should not be relied upon given the small number of patients and ongoing follow-up of the clinical trial. In summary, the study cohort of heavily pre-treated advanced recurrent-staged tumors thus far exhibits an expected high rate of recurrence. The study treatment has been well-tolerated with minimal side effects.

### Tadalafil and MUC1 Vaccine Decrease MDSCs and Treg in the Peripheral Blood and Restore the Expression of CD3 ζ-Chain in the CD8^+^T Cells

**Figure 3:**
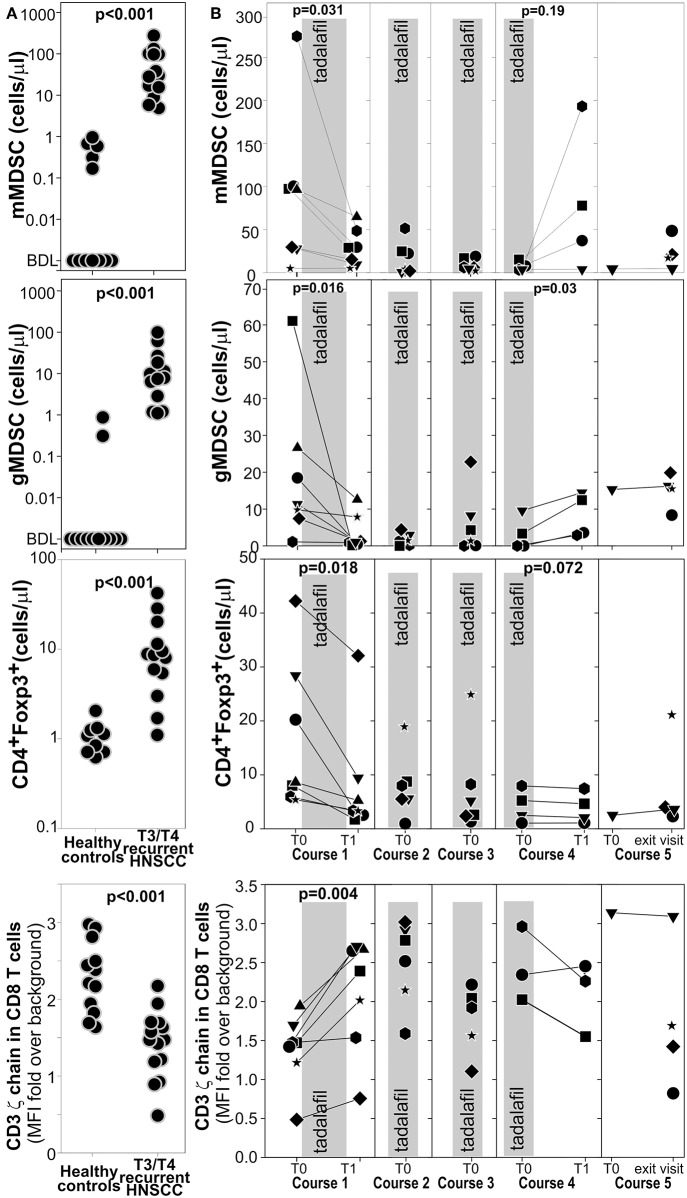
Tadalafil and MUC1/polyICLC vaccine lowers circulating MDSCs and Treg and restores the expression of CD3 ζ-chain expression on CD8 T cells**. (A)** mMDSC, gMDSC, Treg, and the expression of CD3 ζ-chain expression on CD8 T cells was evaluated by multicolor flow cytometry on fresh blood of the enrolled patients or on age matched healthy donors. See Supplementary Figure 3 for gating strategies. Leukocyte subsets were enumerated with “123 beads” Two ways *T*-test value are reported. **(B)** The same subsets as in A were evaluated longitudinally in the patients enrolled in the treatment arm. The gray area correspond to the Tadalafil treatment. Significant Paired *T*-test value are indicated.

Longitudinal Immunomonitoring was performed on the peripheral blood to assess the changes in monocytic MDSC [mMDSC defined as CD33^+^IL4Rα^+^CD14^+^CD11b^+^HLADR^low/−^cells (33)], granulocytic MDSC [gMDSC defined as CD33^+^IL4Rα^+^CD15^+^CD11b^+^HLADR^low/−−^Lox1^+^ cells (50)], and regulatory T cells (Treg defined as CD3^+^CD4^+^Foxp3^+^T cells) (Supplementary Figure 3). Additionally, we evaluated the expression of CD3 ζ-chain in the CD8^+^T cells since its down-regulation is associated with MDSCs activity, T cell apoptosis, disease stage, and worse prognosis in patients with HNSCC (51–53). As expected, compared to age matched healthy controls, mMDSC, gMDSC, and Treg were significantly increased in patients with recurrent stage 3 and 4 HNSCC whereas CD3 ζ-chain in the CD8^+^T cells (Figure 3A) was downregulated.

Even before salvage surgery, Tadalafil treatment (gray shadowed area) significantly lowered both MDSC subsets and Treg and increased the expression of CD3 ζ-chain in the CD8^+^T cells. These positive modulations were maintained during the treatment in course 2, 3, and 4 (Figure 3B). Interestingly, 15 days after Tadalafil termination in course 4, an increased in gMDSCs and mMDSC and a decrease in ζ-chain expression was observed in many patients suggesting that active mechanisms of MDSCs expansion were still present even without any clinical detectable tumor (Figure 3B). Taken together, these results confirm a beneficial action of Tadalafil and possibly MUC1/polyICLC vaccine to the tumor macro-environment. However, these effects are reverted upon treatment discontinuation, possibly suggesting the presence of a microscopic disease being present prior to its becoming clinically evident.

**Figure 4:**
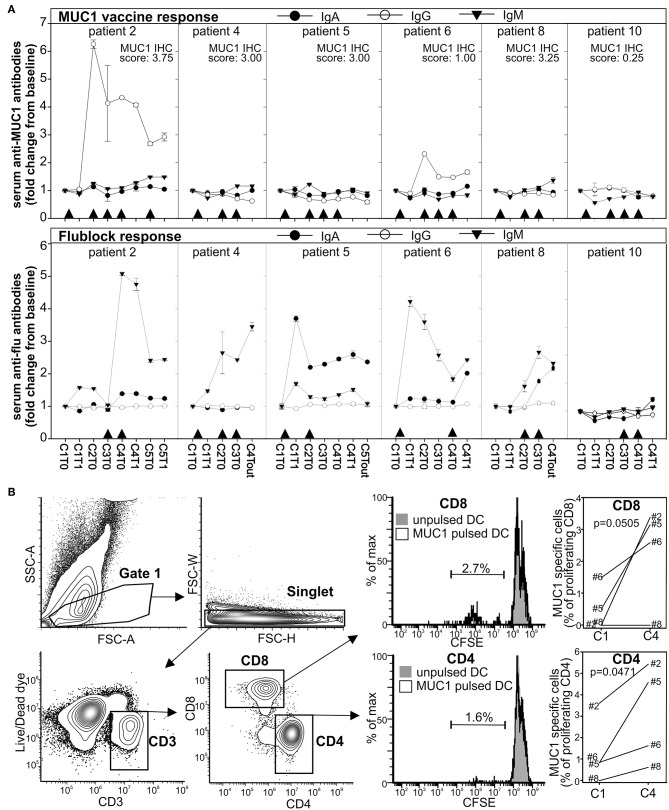
Immune response to the MUC1 and the influenza vaccines. **(A)** Anti-MUC1 or anti-flublock antibodies were evaluated longitudinally by ELISA in the plasma of the patients in the treatment arm. Arrowheads indicate the immunization time. MUC1 IHC score is indicated. **(B)** T cells from PBMCs drawn at baseline (before treatment initiation of course 1) and 2 weeks after completion of course 4 were stimulated with monocytes-derived autologous DC pulsed with MUC1 peptide. Four days later, CD8^+^ T-cell proliferation was evaluated by FACS. Background from parallel culture using unpulsed DC was subtracted.

We then evaluated the capacity of the patients to mount an immune response to tumor associated antigen (MUC1) or unrelated antigens (recombinant flu antigens, flublock vaccine). To accomplish this aim patients that enrolled in the treatment arm received the MUC1 peptide vaccine admixed to polyICLC as adjuvant (intramuscular in the right arm) and the flublock vaccine (intramuscular in the left arm when seasonally available) on day 7 of course 1, on day 10 of course 2, 3 and 4, and on day 0 of course 5. Response to vaccines was evaluated by ELISA on the serum to determine the concentration of IgM, IgG, and IgA against the MUC1 or the influenza antigens. The choice of these assays was determined by their simplicity, HLA type independence, and by the fact that the presence of IgG antibodies against a tumor associated antigen correlates well with the CTL response (54). Longitudinal analysis of the treated patients reveals a higher titer of IgM or IgA antibodies against influenza antigens in 5 out of 6 patients following vaccination (Figure 4A). In contrast, only two patients showed a significant immune response against the MUC1 vaccine (Figure 4A). Interestingly, the responses to the MUC1 vaccine were observed only in the patients that had not received chemotherapy in conjunction with their radiation treatment for their original cancer treatment prior to recurrence.

To evaluate whether treatment could increase T cell mediated immunity against MUC1, magnetically purified CD3^+^ T cells, harvested before treatment initiation (C0) and 2 weeks after course 4 (C4), were stimulated with autologous DCs pulsed with the MUC1 peptide or left unpulsed. CD8^+^ and CD4^+^ T cell proliferation was evaluated 4 days later by flow cytometry (Figure 4B). Compared to baseline, an increase response to the relevant peptide (up to 2–3% of proliferating T cells within the CD4 or CD8 populations) was detected after the 4 courses of treatment in 3 of the 4 evaluated patients.

Taken together these data suggest that Tadalafil and MUC1/polyICLC vaccine positively modulate the immune system systemically in patients with recurrent HNSCC undergoing salvage surgery. However, a strong memory IgG immune response against underglycosylated MUC1 is detectable only in a fraction of the patients.

### Tadalafil and MUC1/polyIC Treatment Lowers MDSCs and Treg at the Tumor Site and Reverse Immune Exclusion

**Figure 5:**
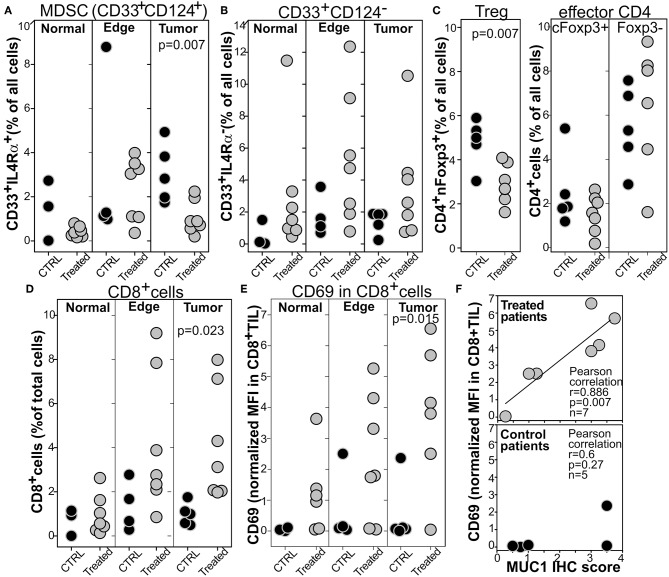
Tumors from patients treated with Tadalafil and MUC1/polyICLC vaccine show a lower infiltration of MDSCs and Treg and a higher infiltration of activated CD8 in the tumor bed. Computer based image cytometry was performed to enumerate the number of **(A)** MDSC, **(B)** IL4Rα^−^myeloid cells, **(C)** CD4^+^T cell subsets, or **(D)** CD8^+^T cells. **(E)** CD69 expression within the CD8 is reported normalized on the CD69 expression on all the cells evaluated. Depending on the region of interest evaluated, at least 10^5^-10^6^ cells were analyzed. **(F)** The expression of CD69 in CD8^+^T cells was plotted against MUC1 IHC score of the corresponding tumor. Two ways *T*-test and relevant pearson correlation parameters are reported.

Immunofluorescence based image cytometry was employed to determine the effect of treatment at the tumor site both objectively and topographically (Supplementary Figure 4). Briefly, slides from the surgical specimens were stained to identify CD33^+^IL4Rα^+^MDSCs (33), CD4^+^Treg expressing Foxp3 in the nucleus (55), or activated CD8^+^CD69^+^ T cells. Stained slides were acquired with a high resolution microscanner, processed with cell-profiler to identify each individual cell, and fed into FCS-image express to enumerate the cell of interest and the expression of a particular protein. This process allowed for analysis of 10^5^-10^6^ cells inside the tumor, at the tumor edge, and in “normal” adjacent tissue as defined by an experienced pathologist in serial H&E slides. Compared to the untreated controls, CD33^+^IL4Rα^+^MDSCs were significantly lower inside the tumor in the treated patients (Figure 5A), whereas no differences were found at the tumor edge or in the “normal” tissue nor in the total number of CD33^+^IL4Rα^−^ myeloid cells (Figure 5B and Supplementary Figure 5). Similarly, a lower concentration of Tregs with nuclear FoxP3 was found in the tumor of treated patients compared to controls whereas no differences were detected in the naïve (CD4^+^Foxp3^−^cells) or poorly activated [CD4^+^cells with cytoplasmic Foxp3 (55, 56)] CD4^+^T cells (Figure 5C). Conversely, a higher number of CD8^+^T cells were found in the tumor of treated patients compared to the controls whereas no differences were found in the tumor edge and in the surrounding normal tissue (Figure 5D and Supplementary Figure 4E). Furthermore, analysis of CD69 indicated a significantly higher expression of this early activation marker in the CD8^+^cells (Figure 5E and Supplementary Figure 4F) of the treated patients compared to controls. Interestingly, the expression of CD69 significantly correlates with the MUC1 expression in the same specimen determined by IHC in the treated patients whereas no correlation was observed in the untreated controls (Figure 5F). This suggests that despite the poor immune response against MUC1 detected in most patients (Figure 4), an immune response against this tumor associated antigen has been primed and resulted in the infiltration of activated CTL at the tumor site.

Taken together, these results indicate that Tadalafil and polyICLC/MUC1 vaccine reshape the tumor microenvironment, lowering the immune suppressive populations and increasing the number of activated T cells.

### Reversion of Immune Exclusion by Tadalafil and MUC1/polyICLC Vaccine Promotes PDL1 Expression on CD163^−^ Cells

**Figure 6:**
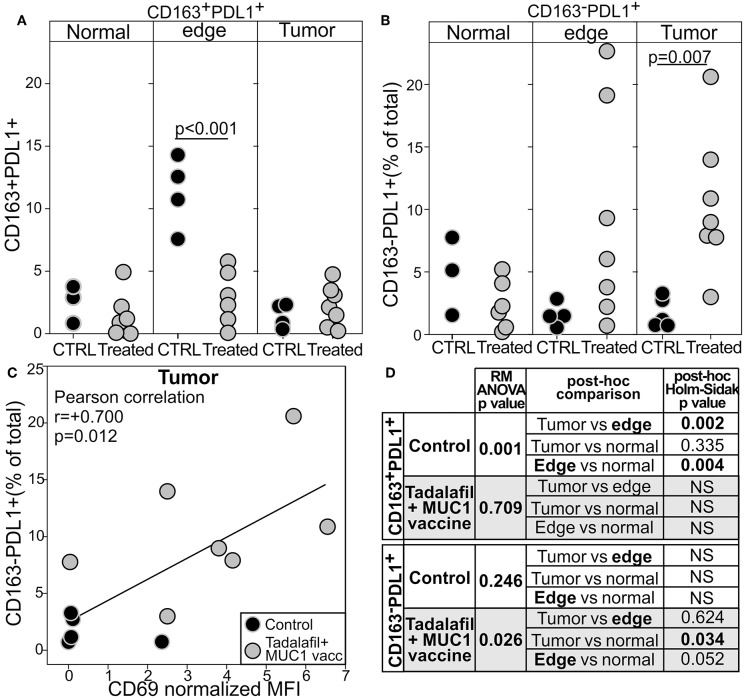
Tadalafil and MUC1/polyICLC vaccine treatments modulate the expression of PDL1 in the tumor microenvironment. The expression of PDL1 within the CD163+ **(A)** or the CD163^−^cells **(B)** was quantified by image cytometry in the tumor, at the tumor edge, or in “normal” surrounding tissue in the tumor specimen from the control (black filled circle) or Tadalafil and MUC1/polyICLC vaccine treated (gray filled circle) patients. Two way *T*-test *p*-value are reported. **(C)** Correlation between the expression of CD69 in the CD8^+^T cells and PDL1 expression on the CD163^−^cells. **(D)** Summary of the one way RM ANOVA analysis.

Notwithstanding the limited number of treated patients, and despite the positive changes in the tumor microenvironment, the priming of an immune response against a tumor associated antigen, and the removal of all the tumor mass by salvage surgery, we did not observe a dramatic reduction of tumor recurrence in this high risk population (Supplementary Figure 6). We thus evaluated whether the higher number of activated T cells in the tumor may elicit additional mechanisms of immune escape. Indeed, in HNSCC as well as in other malignancies IFNγ released by CD8^+^T cells was shown to upregulate PDL1 on neoplastic cells (57–59). We thus evaluated the expression of the checkpoint molecule PDL1 and the macrophage marker CD163 by image cytometry in the tumor of the enrolled patients. In the control untreated patients, PDL1 was mostly confined in CD163^+^macrophage at the tumor edge whereas tumor and normal surrounding tissues expressed low level of this protein (Figure 6A and Supplementary Figure 7). Conversely, in the treated patients CD163^+^PDL1^+^macrophages were significantly lower in the tumor edge and at concentration levels similar to that of the surrounding tissue (Figure 6A and Supplementary Figure 7). However, PDL1 expression at the tumor edge of the treated patients did not differ from that of the control patients because of an increase of this inhibitory marker in the CD163^−^cells (Figure 6B). Indeed, the intratumoral expression of PDL1 was significantly higher in treated patients than in the control patients and confined mostly in the CD163^−^ cells (Figure 6B and Supplementary Figure 7). Interestingly, the level of expression of PDL1 directly correlated with the expression of CD69 on the tumor infiltrating CD8^+^cells, suggesting the instauration of a T cell dependent mechanism of PDL1 upregulation and immune escape (Figure 6C).

**Figure 7:**
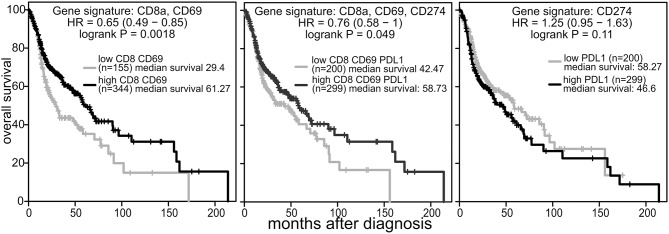
PDL1 expression limits the beneficial prognostic value of CD8a and CD69 in the tumor. KM plotter analysis (kmplot.com) was performed on tumor RNAseq data from patients with HNSCC (*n* = 499, all tumor stages) using the mean expression of the indicated genes with weight =1 and auto select best cutoff selected.

These data suggest that the beneficial activity of Tadalafil and MUC1 vaccine might be hindered by this corresponding upregulation of PDL1. Indeed, KMplotter analysis [KMplot.com, (60)] on RNAseq data from 499 patients with HNSCC reveals that the expression of CD8a and CD69 mRNA in the tumor well-correlate with improved survival (Figure 7). Addition of PDL1 (CD274) to the gene signature interrogated, drastically reduced the benefit of a higher infiltration in the tumor of CD8^+^CD69^+^ cells. No negative effects were noted when only expression of CD274 was evaluated.

## Discussion

This phase 1 clinical trial, designed in patients with recurrent HNSCC undergoing salvage surgery to evaluate the safety of combining chronic PDE5 inhibition to reverse tumor-induced immunosuppression, and MUC1/polyICLC immunization to prime a tumor specific immune response, seems to confirm the previous clinical evidence indicating beneficial actions for these two interventions in patients with malignancies. Indeed, initial case reports indicate an antitumor activity of PDE5 inhibition in patients with Waldenstrom's macroglobulinemia (61), B-cell chronic lymphocytic leukemia (62), and penile cancer (63). Tadalafil was successfully used to treat a patient with end-stage relapsed/refractory multiple myeloma (64), generating a dramatic and durable anti-myeloma immune response and clinical response with associated transfusion independence and improvement in quality of life (64).

Clinical trials are being performed in colorectal cancer (NCT02998736), Glioma (NCT01817751), abdominal malignancies (NCT02998736), advanced solid tumors (NCT02466802), pancreatic cancer (NCT01342224), myelodysplastic syndrome (NCT03259516), multiple myeloma (NCT01858558), metastatic melanoma (EudraCT-No: 2011-003273-28), and Head and Neck squamous cell carcinoma (NCT00843635, NCT00894413, NCT01697800, NCT03238365, NCT02544880). To date, data are available only for our two previous clinical trials in HNSCC and for a dose escalating open label clinical trial in progressive metastatic melanoma (65). In these trials, chronic tadalafil treatment lowered MDSCs and Treg in the blood and at the tumor site (32, 33, 65), restored the immune response to recall antigens (32), enhanced the expression of CD3 ζ-chain in CD8^+^T cells (32), primed/enhanced the tumor specific immune response, and increased the number of tumor infiltrating T cells (33). However, notwithstanding the low number of patients enrolled in these trials, the positive immunomodulatory actions of PDE5 inhibition were associated with no clinical benefits (Supplementary Figure 1), although disease stabilization was reported for few patients in the melanoma trial and in case reports (64, 65).

Most of the beneficial immunomodulations of Tadalafil are confirmed in our ongoing phase 1 clinical Trial. Indeed, treatment was associated with a reduction of mMDSC and Treg in the blood and at the tumor site, an increase in the expression of CD3ζ chain at the tumor site and a higher infiltration of activated CD8^+^ T cells at the tumor site (Figures 3, 5).

After confirming MUC1 as a tumor specific antigen in recurrent HNSCC (Figure 1), for the first time we evaluated the safety and immunological potential of combining the MUC1 peptide/polyICLC vaccine with Tadalafil treatment. Despite finding a detectable IgG immune response in only 2 of the 6 treated patients (Figure 4A), the combined treatment seems to increase T cell reactivity to MUC1 (2–3% of T cell proliferation within the CD4 or CD8 gates to the relevant peptide) in most of the evaluable patients after 4 treatment courses (Figure 4B), and a significant correlation was found between the activation of tumor infiltrating lymphocytes and MUC1 expression in the tumor (Figure 5). Notwithstanding the low number of patients evaluated, taken together, these data suggest a possible priming of MUC1 immunity in most patients with MUC1^+^tumors. Notwithstanding the low number of patients evaluated, taken together, these data suggest a possible priming of MUC1 immunity in most patients with MUC1^+^tumors.

The combined treatment of Tadalafil and MUC1 vaccine was well-tolerated with no serious side effects, and no treatment limiting toxicity observed. One subject was withdrawn from the study for the development of an asymptomatic autoimmune disorder as determined by the detection of anti-nuclear antibodies while on treatment. Subsequent ANA testing in this subject did revert to normal, with no clinical signs of autoimmunity detected at any time during treatment or after treatment discontinuation. The ANA test was selected as a screening tool for autoimmune disease for this trial, with a positive ANA test considered an exclusion criteria for enrollment. It should be noted that a total of 5 subjects otherwise eligible for enrollment in the phase I trial were excluded because of an asymptomatic positive ANA. While the study subject's development of a positive ANA while on treatment was interpreted as a potential sign of a treatment induced asymptomatic autoimmune disorder, and the subject was withdrawn from further treatment accordingly, this significant incidence of asymptomatic positive ANA in this patient cohort also raises the possibility that this finding may have been unrelated to the study intervention. Regardless, the combined immunologic interventions of this trial did not result in any clinically symptomatic autoimmune disease.

While this phase I study was not designed to demonstrate clinical efficacy of the study drug combination, the very poor prognosis and the expected high recurrence rate of the patient cohort studied provided for the potential identification of clinical efficacy should a dramatic clinical effect be demonstrated. Despite complete surgical extirpation of tumor and the addition of PDE5 inhibition and MUC1/PolyICLC vaccination, however, no such dramatic clinical benefit was detected (Supplementary Figure 6). This prompted us to evaluate whether additional mechanisms of immune escape were induced after reversal of immune exclusion.

Evaluation of PDL1 expression on macrophages and on CD163 negative cells at the tumor site via image cytometry suggest that while Tadalafil and/or polyICLC vaccine are effective in reducing PDL1^+^macrophage at the tumor edge, the increase of CD69^+^T cells within the tumor promotes (Figure 5) the expression of this inhibitory molecules on CD163^−^cells (Figure 6). Indeed, a prominent role of activated T cells secreting type 2 interferon is emerging as inducer of PDL1 in neoplastic cells (57–59). For example, cisplatin and IFNγ have been shown to upregulate PDL1 on cell lines of HNSCC (66) and the secretion of this cytokine by activated CTL at the tumor site play a key role in the upregulation this checkpoint molecules in gastric cancer cells (67). In line with these observations we did find an intriguing correlation between the expression of CD69 in CTL at the tumor site and the expression of PDL1 (Figure 6).

It is important to note that our phase 1 study is limited by the low number of patients enrolled, by the open label single arm design among the treated patients, by the absence of randomization between the control and treated patients, and by the fact that the design of this phase 1 lead-in clinical trial does not allow for the discrimination between the immunological effects of Tadalafil and the immunological effects of the MUC1/polyICLC vaccine. Notwithstanding these limitations, however, to our knowledge this trial provides the first evidence that the combination of Tadalafil and the anti-MUC/polyICLC vaccine can reverse immune exclusion but also promote the upregulation of PDL1 as additional mechanisms of tumor escape. The notion that PDL1 upregulation may limit the efficacy of Tadalafil and vaccine based immunotherapy is further supported by the analysis of public RNAseq database. These analysis indicates that the beneficial prognostic role of the CD8CD69 signature in HNSCC is partially decreased by PDL1 expression.

Taken together, the interim analyses of this phase 1 clinical trial indicate that the treatment combination is safe and well-tolerated, can reverse immune exclusion, but can also promotes PDL1 upregulation. The latter finding provides a mechanism by which the proposed treatment combination may have offsetting immunologic outcomes. As such, a decision has been made to suspend accrual to the randomized phase II trial as designed given this potential limitation in experimental treatment efficacy. Instead, a new combinatorial intervention is being explored that conjugates salvage surgery, inhibition of PDE5, priming of an anti-tumor immune response, and checkpoint inhibition.

## Data Availability

All datasets generated for this study are included in the manuscript and/or the Supplementary Files.

## Ethics Statement

This study was carried out in accordance with the recommendations of the Protocol Review Committee of the Sylvester Comprehensive Cancer Center of the University of Miami Miller School of Medicine and as reviewed and approved under IND 16403 by the Food and Drug Administration, with written informed consent from all subjects. All subjects gave written informed consent in accordance with the Declaration of Helsinki. The protocol was approved by the Institutional Review Board of the University of Miami.

## Author Contributions

DW designed the study, oversaw the clinical trial and all the regulatory aspects, performed the salvage surgery, wrote the paper and interpreted the data. SZ performed the flow and image cytometry experiments and analyzed the data. IR provided the statistical support and analyzed the data. ZS and FC helped with patient enrollments and performed salvage surgery. MA helped with the patient follow up of our former clinical trial and with initial observation of PDL1 expression. CG-F provided the pathologic expertise. CR helped with blood processing and IHC analysis. PS designed the study, the immune monitoring assays, analyzed and interpreted the data and wrote the paper.

### Conflict of Interest Statement

PS is named as inventor in patent owned by Johns Hopkins University regarding the use of PDE5 inhibitors as immune modulator. The remaining authors declare that the research was conducted in the absence of any commercial or financial relationships that could be construed as a potential conflict of interest.
