# Supplementary material for: The Reversal of Immune Exclusion Mediated by Tadalafil and an Anti-tumor Vaccine Also Induces PDL1 Upregulation in Recurrent Head and Neck Squamous Cell Carcinoma: Interim Analysis of a Phase I Clinical Trial
Source: Front Immunol. 2019 May 31;10:1206. doi: 10.3389/fimmu.2019.01206 (PMC6554471; doi:10.3389/fimmu.2019.01206)
Supplement: Supplementary file 1 [file Data_Sheet_1.pdf]

Supplementary material

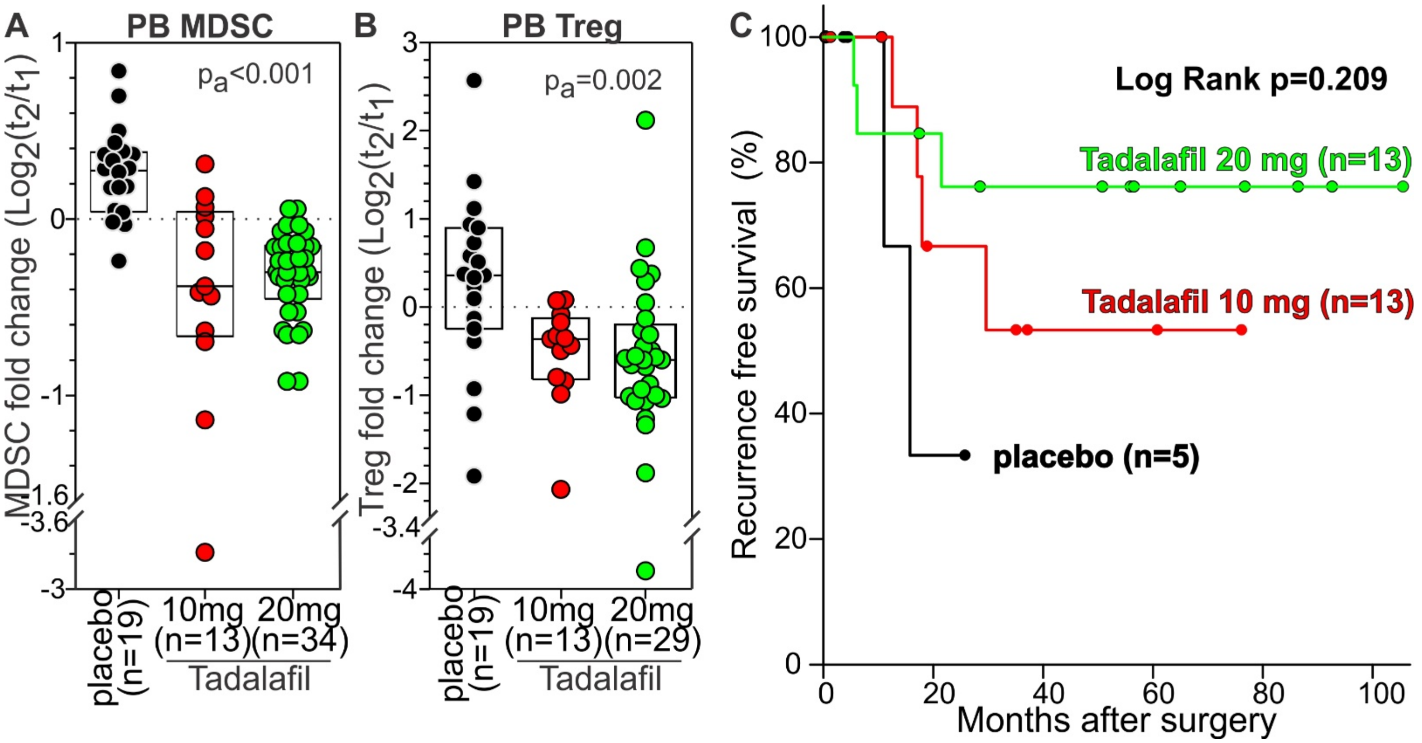

Supplementary figure 1: Tadalafil in a neoadjuvant setting reduces MDSCs and Treg in the blood of patients with HNSCC but a modest long-term clinical effect. A meta-analysis was performed of our two previous clinical trials (NCT00843635 and NCT00894413) to evaluate the modulation of MDSC **(A)** and Treg **(B)** in patients with primary HNSCC randomized to be treated with placebo, or Tadalafil (10 or 20 mg) before surgery. **C)** Kaplan Meyer plot and Log Rank analysis of the recurrence free survival of the patients enrolled in the (NCT00843635) trial.

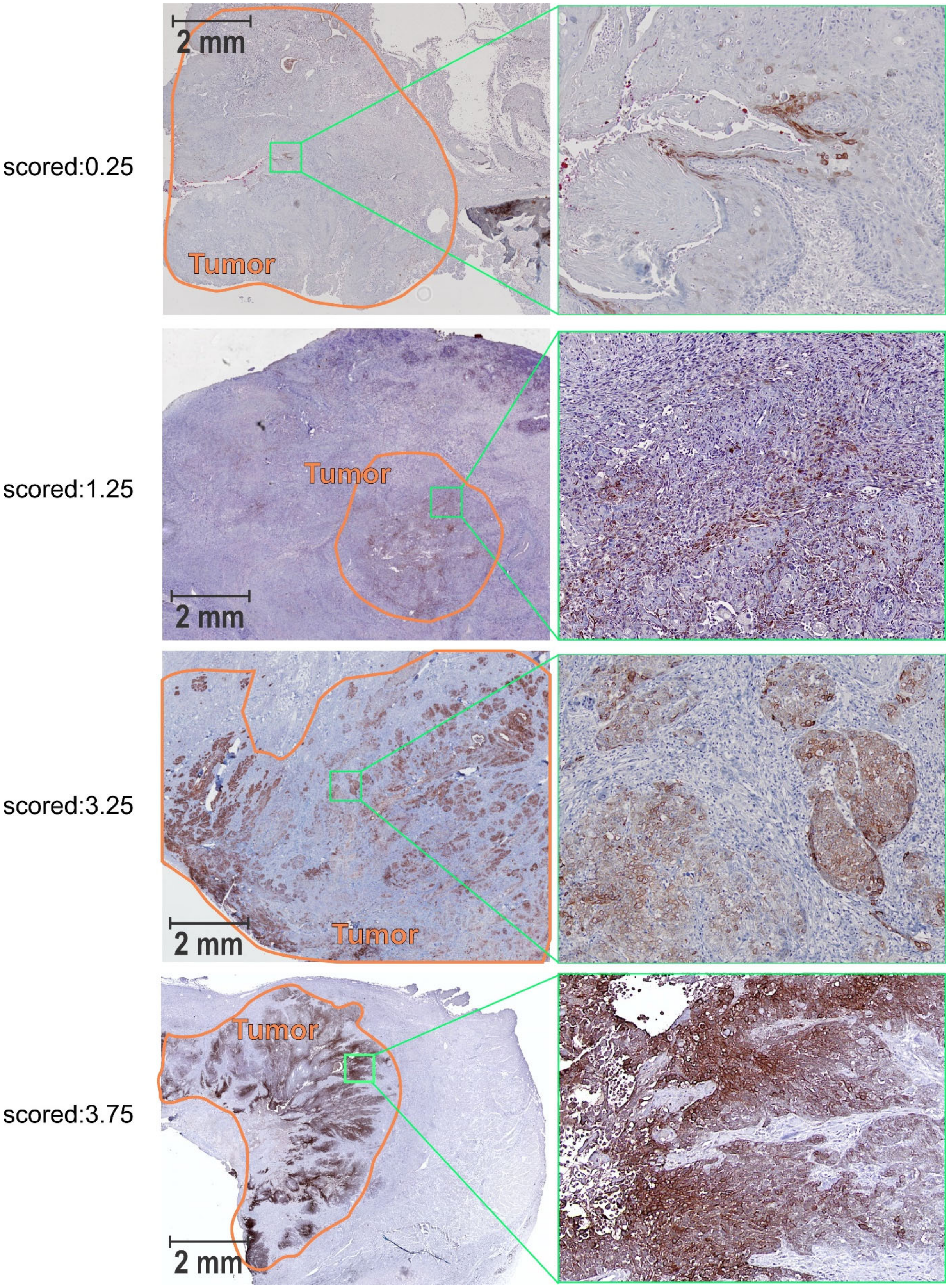

Supplementary figure 2: Examples of IHC for underglycosylated MUC1 and relative scores.

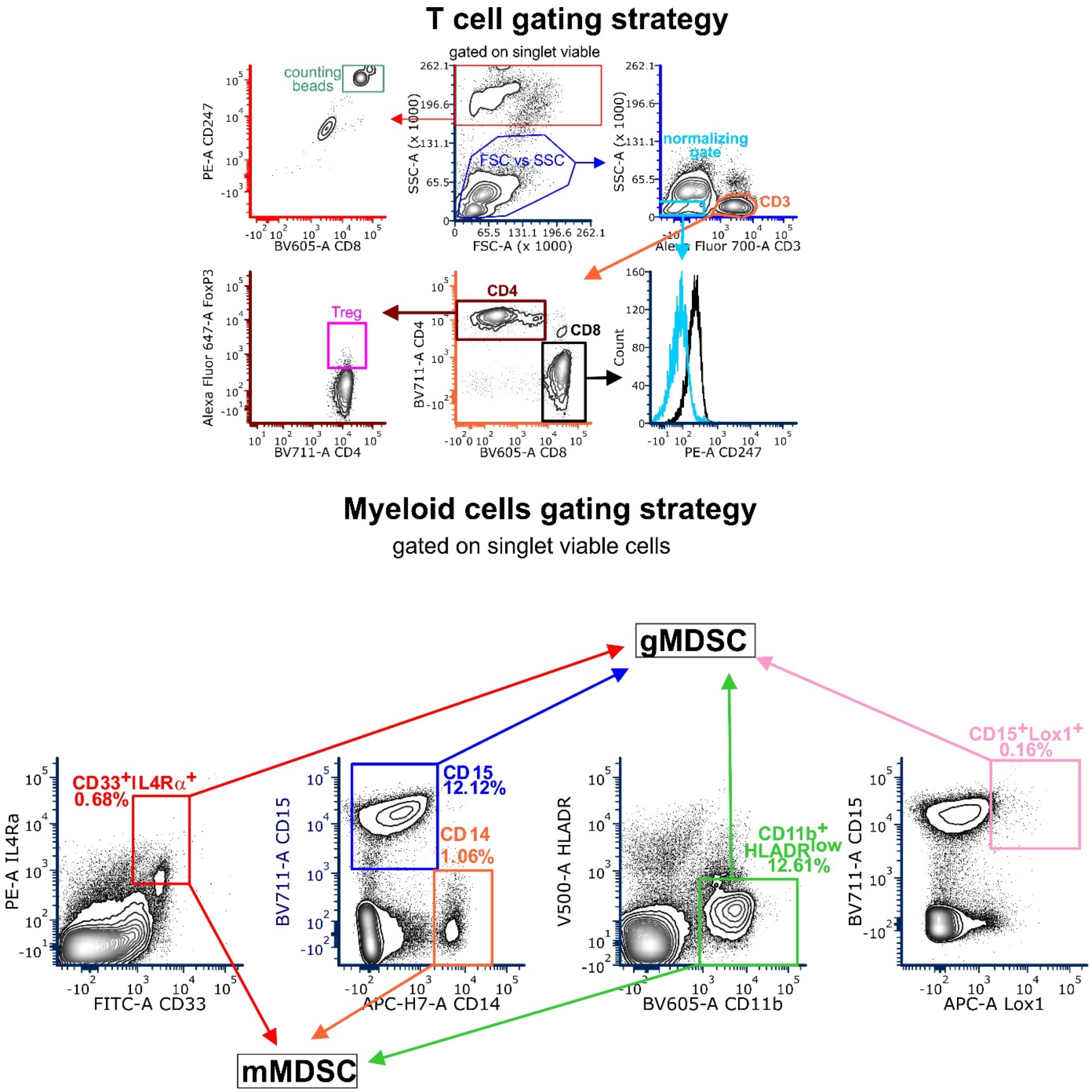

Supplementary figure 3: Gating strategies for the flow cytometry based enumeration of T cell and MDSC subsets. To compare the expression of CD3 ζ-chain, CD247 MFI in CD8^{+}T cells was normalized with that of the singlet viable CD3 negative SSCA^{low} cells (Cyan "normalizing gate")

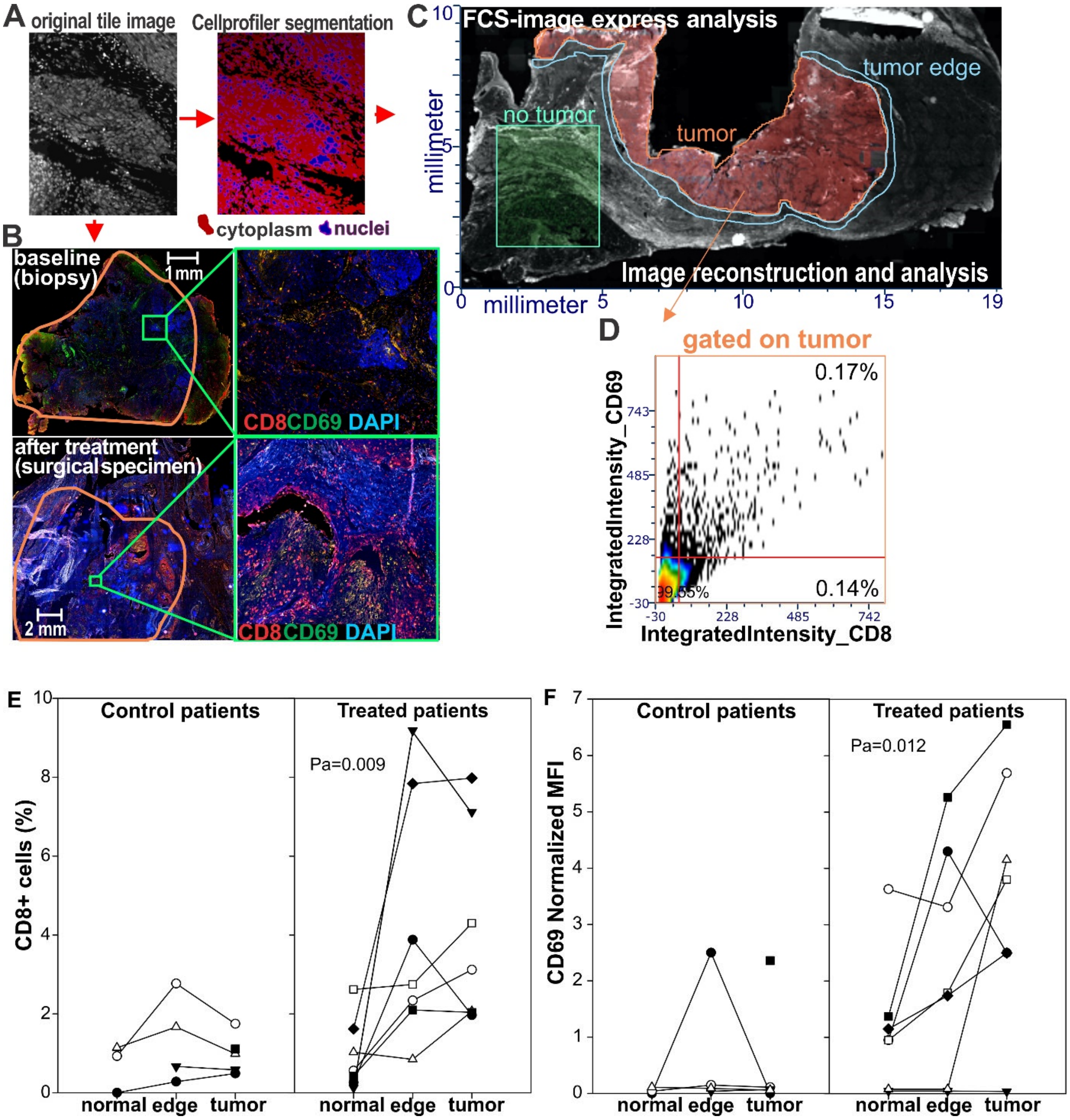

Supplementary figure 4: Tadalafil and poly-ICLC/MUC1 increase the number of activated CD8 in the tumor. Tumor specimens were labeled with antibodies against CD8 and CD69 and counterstained with DAPI. Images were acquired with the Olympus vs120 at 20X magnification. Tile images (A) were stitched as in the examples (B) of tumors before (biopsy) or after treatment (surgical specimen) of one of the patient. Stitched images were process with cell profiled and fed into FCS-image express (C) for data image cytometry analysis using tools similar to the one available in flow cytometry. D) Density plot of the cells gated in "tumor region" of a patient treated with MUC1/polyICLC and Tadalafil. E, F) Stained slides from control and treated patients were scanned and CD8+T cell infiltration and activation (CD69) was quantified by image cytometry after gating in the "tumor region" or in "no tumor area" indicated in serial H&E by an experienced HNSCC pathologist.

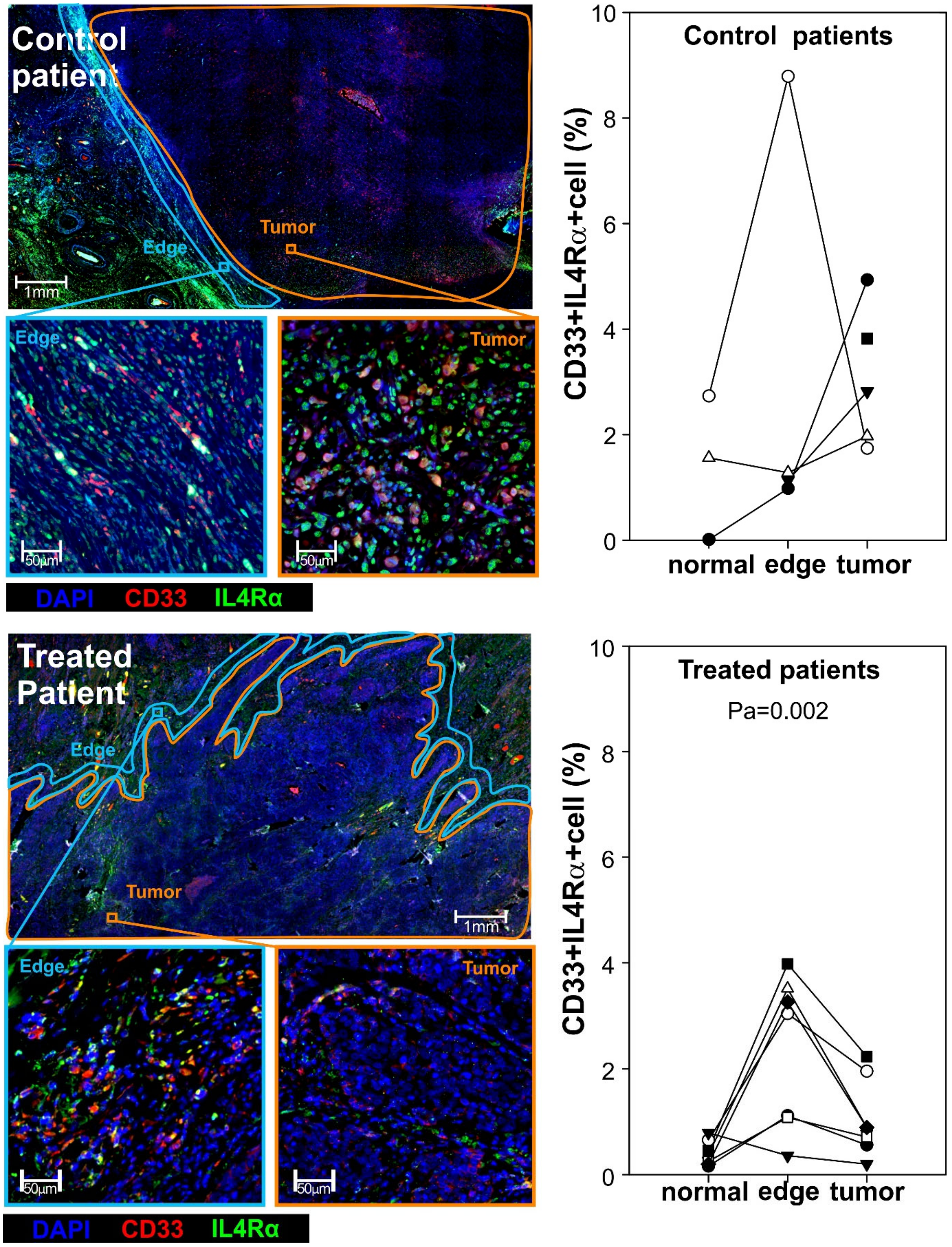

Supplementary figure 5: Example of CD33 and IL4Ra staining on control and treated patients. Image cytometry was performed as described in Fig.5 and data are reported as spaghetti plot across the normal tissue, tumor edge area or inside the tumor. RM-one way ANOVA p value is reported.

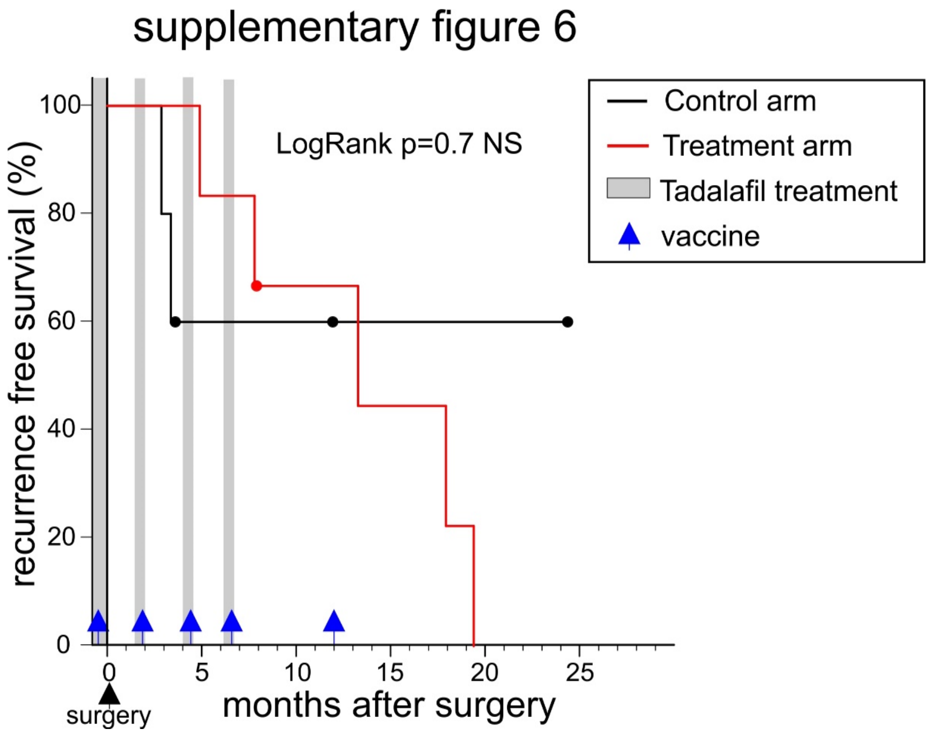

Supplementary figure 6: Interim recurrence free-survival logrank analysis of the patients enrolled in the ongoing trial. Time to recurrence is defined as the interval from the time of the surgery and the time in which recurrent tumors were detected. Tadalafil treatment is highlighted by the grayed area, and immunization time by blue arrows.

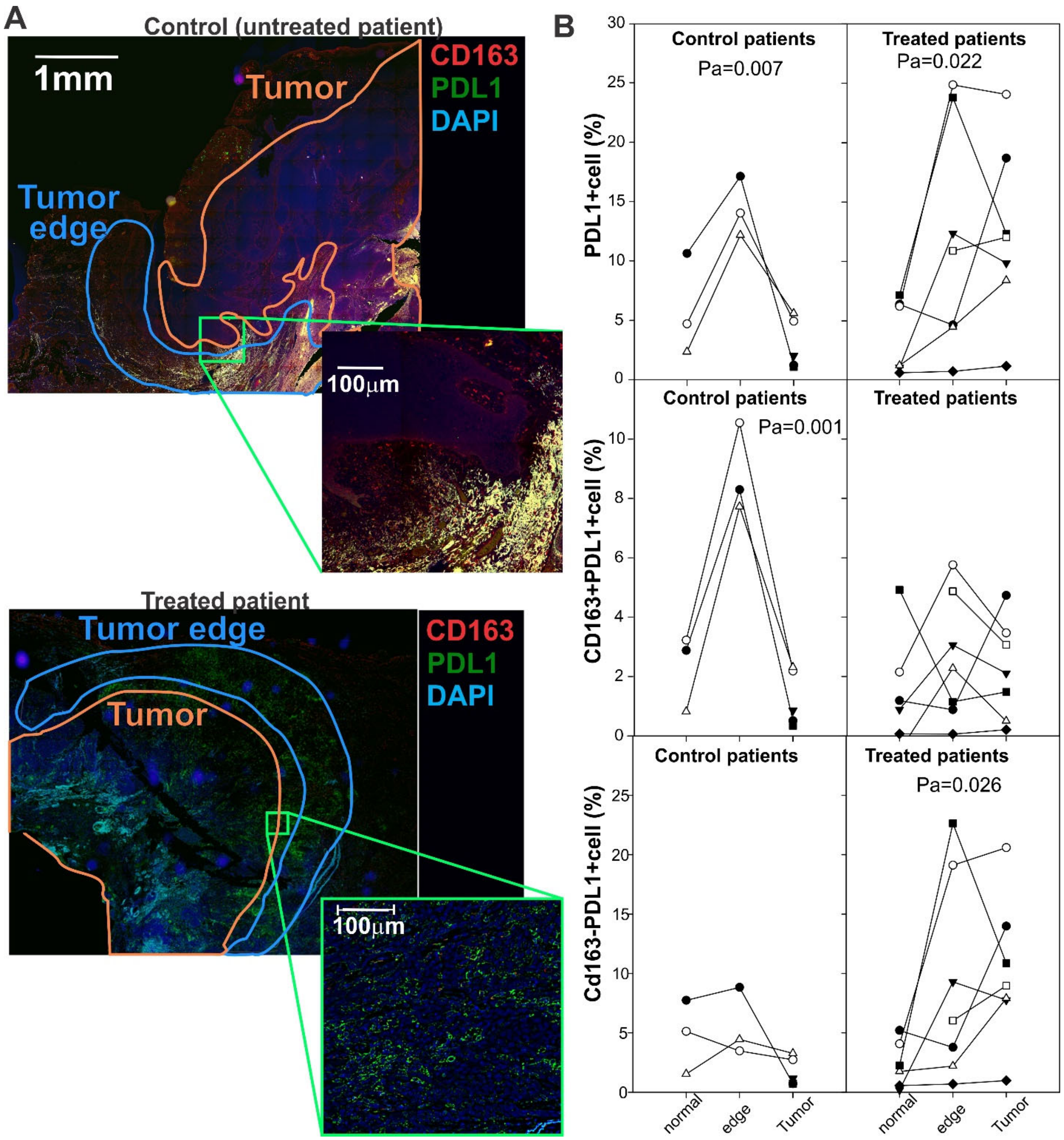

Supplementary figure 7: Example of CD163 and PDL1 staining on control and treated patients. Image cytometry was performed as described in Fig.5 and data are reported as spaghetti plot across the normal tissue, tumor edge area or inside the tumor. RM-one way ANOVA p value is reported.

Supplementary methods:

Image cytometry and image processing

Tiff files from whole slide scan were optimized with imageJ and sliced in 600x600 pixel images using ImageSlicer (https://www.coolutils.com/TotalImageSlicer) each identifiable as metadata for column and row. Sliced images were loaded in cell profiler, converted to a gray scale. Nuclei were identified as the primary object using the blue (DAPI) channel by setting the diameter of the nuclei between 2 and 10 pixels, using the three classes Otsu Adaptive threshold method with a correction factor of 1 and the lower and upper bounds on threshold 0.1–1.0. Clumped objects were distinguished by shape and the size of the smoothing filter and minimum allowed distance between local maxima were automatically calculated. Secondary objects (i.e. cells) were identified using the auto-fluorescence and fluorescence of the merged image from the 3 channels acquired using the nuclei propagation method with three classes Otzu Adaptive threshold method, 0.9 as threshold correction factor (0.0–1.0 range) and 0.02 as regularization factor. The cytoplasm as tertiary object was identified as the area included in the cells (secondary object) but not in the nuclei (primary object). For each cell, integrated intensity mean of the blue channel (DAPI) in the nuclei, integrated intensity mean of the green (PD-L1; CD33 or CD69) and red (CD163; IL4Ra or CD8) channel of cells and cytoplasm were exported as .cpout file. Cpout files were analyzed using FCS Express 6 PLUS. Areas of interest corresponding to the neoplastic lesion of the tissue were identified by an experienced pathologist in serial section stained with H&E. Images from FoxP3 and CD4 staining was processed with the procedure described above and analyzed as previously reported (Weed et al., 2013).

Inclusion and exclusion criteria

Inclusion Criteria

1. Biopsy-proven recurrent or second primary HNSCC of the oral cavity, oropharynx, hypopharynx or larynx (second primary includes unknown primary)
2. Stage III or IV (AJCC, 7th ed., 2010) recurrent or second primary HNSCC (For recurrent tumors, staging is determined by the recurrent stage, not by the original pretreatment stage.)
3. Surgically resectable, recurrent or second primary HNSCC
4. Prior radiation with or without prior surgery and/or chemotherapy, to the head and neck for definitive treatment of HNSCC of the oral cavity, oropharynx, hypopharynx or larynx with previously documented complete clinical or radiographic response to initial treatment
   a. Prior radiation and any chemotherapy, must have been completed >4 months prior to biopsy-proven recurrence or second primary site disease
   b. Recurrent or second primary HNSCC arises within the previously irradiated field
5. Age ≥18 years
6. Eastern Cooperative Oncology Group (ECOG) Performance Status of 0, 1 or 2 or equivalent scale score. See Appendix D for equivalent scale criteria.
7. Acceptable organ function as defined by all of the following:
   - Alkaline phosphatase < 4.0 x ULN
   - AST ≤ 2.5 x ULN
   - ALT ≤ 2.5 x ULN
   - calculated Creatinine Clearance ≥ 51ml/min as determined by the *Cockcroft-Gault Equation*: [(140-age) * (Weight in kg) * (0.85, if female)] / (72 * Cr)
8. Suitable venous access to allow for all study related blood sampling (safety and research)
9. Ability to understand and willingness to sign the written informed consent and HIPAA document(s).

Exclusion Criteria

1. Salvage surgery is not recommended as per NCCN guidelines, or after multidisciplinary treatment evaluation, including those with surgically unresectable disease at primary site or regional lymph nodes.

2. Recurrent or second primary AJCC Stage I or II HNSCC (for recurrent tumors, staging is determined by the recurrent stage, not by the original pretreatment stage).
3. Distant metastatic disease.
4. Recurrent or second primary HNSCC of the nasopharynx, paranasal sinuses, or cervical esophagus.
5. Use of PDE5 inhibitors such as vardenafil (Levitra®), Tadalafil (Cialis®), and sildenafil citrate (Viagra®) ≤15-days prior to (intended) enrollment.
6. Patients who have the intention to receive non-study PDE5 inhibitors and flu vaccination(s) anytime during the study will be excluded.
7. Prior or known adverse reactions to PDE5 inhibitors, poly-ICLC (Hiltonol®), and prior dose(s) of Influenza vaccine including but not limited to their components.
8. History of severe or unstable cardiac or cerebrovascular disease:
    a. Myocardial infarction within the last 90 days.
    b. Unstable angina or angina occurring during sexual intercourse.
    c. New York Heart Association (NYHA) Class 2 or greater heart failure in the last 3 months (See Appendix E).
    d. Uncontrolled arrhythmias.
    e. Sustained hypotension (<90/50 mmHg) or uncontrolled Hypertension (>170/100 mmHg).
    f. Stroke within the last 6 months.
9. Therapy with nitrate, alpha-blocker, or cytochrome P450 (CYP3A4) inhibitors within 7-days prior to study treatment initiation and for whom stopping is unsafe and/or a safe substitute is not medically recommended. Some examples are provided in Appendix A.
10. Positive Antinuclear Antibody Test (ANA).
11. Immunosuppression or immunocompromised for reasons not directly related to patient's malignancy (e.g. HIV or kidney transplant).
12. History of severe or life threatening autoimmune diseases [Exceptions: Mild autoimmune diseases determined at the discretion of the Investigator(s), e.g. psoriasis.].
13. Unilateral blindness, hereditary retinal disorders, or at an increased risk of blindness
14. Unilateral deafness, or severe hearing loss dependent upon hearing aid(s) for serviceable communication.
15. Female patients who are pregnant or breastfeeding. (Females of childbearing potential are required to have a negative urine β-human chorionic gonadotropin (β-hCG) pregnancy test result obtained during screening; pregnancy testing is not required for post-menopausal or surgically sterilized women).
16. Females of childbearing potential who refuse to practice effective methods of contraception or abstain from heterosexual intercourse from the time of signing the informed consent through 30-days after the last vaccination.
17. Serious medical or psychiatric illness/condition, including alcohol or drug abuse likely in the judgment of the Investigator(s) to interfere with compliance to protocol treatment/research.
18. Patients of vulnerable populations such as children less than 18 years of age, prisoners, institutionalized individuals or others likely to be vulnerable are not eligible for participation in this study.

Supplementary Table 1. Patient demographics

	All Patients		Phase 1		Control		
	N	%	N	%	N	%	P
Total Patients	14	100.0	8	100.0	6	100.0	
Age, in years							
Mean (SD)	63	(6.3)	64.9	(4.2)	60.5	(8.1)	0.21
Median (Min-Max)	64	(49-72)	66.5	(58-71)	60.5	(49-72)	
Gender							
Female	2	14.3	1	12.5	1	16.7	1.00
Male	12	85.7	7	87.5	5	83.3	
Race / Ethnicity							
White / Not Hispanic or Latino	11	78.6	7	87.5	4	66.6	0.69
White / Hispanic or Latino	1	7.1	-	-	1	16.7	
Asian / Not Hispanic or Latino	2	14.3	1	12.5	1	16.7	
ECOG at baseline							
0	-	-	4	50.0	-	-	

Supplementary Table 2. AEs grade and attribution per patient and by type

Pt #	Patient Id / TLT	Total AEs	Baseline	SAE (7)			AE(16)					Late AE (3)
			G2 (1)	G3 (6)		G4 (1)	G1 (9)			G2 (7)		G5 (3)
			Unrelated	Unlikely	Unrelated	Unlikely	Probable	Possible	Unlikely	Probable	Unlikely	Unrelated
		N	N	N	N	N	N	N	N	N	N	N
	All	26	1	3	3	1	5	1	3	2	5	3
**1.01**	Not Evaluable	8	-	2	-	1	-	-	-	-	4	1
**1.02**	No TLT	1	-	-	-	-	-	-	1	-	-	-
**1.04**	No TLT	3	-	-	-	-	-	1	2	-	-	-
**1.05**	No TLT	4	-	1	2	-	-	-	-	-	1	-
**1.06**	No TLT	1	-	-	-	-	-	-	-	-	-	1
**1.08**	No TLT	4	-	-	-	-	1	-	-	2	-	1
**1.09**	Not Evaluable.	4	-	-	1	-	3	-	-	-	-	-
**1.10**	No TLT	2	1	-	-	-	1	-	-	-	-	-

Supplementary Table 3.

Treatment –related AEs grade and attribution per patient and by type

Pt #	Patient Id / TLT	Total AEs	Type/ Grade / Attribution		
			AE		
			1		2
			Probable	Possible	Probable
		N	N	N	N
	**All**	**8**	**5**	**1**	**2**
1.04	No TLT	1	-	1	-
1.08	No TLT	3	1	-	2
1.09	Not Evaluable	3	3	-	-
1.10	No TLT	1	1	-	-

Treatment-related AEs (definite, probable, possible): N=8

- Autoimmune disorder: 1 grade 1 possible (Id 1.04)
- Flushing: 1 grade 1 probable (Id 1.08)
- Headache: 2 grade 2 headache probable in one patient (Id 1.08), 1 grade 1 probable AE (Id 1.09)
- Myalgia: 1 grade 1 probable (Id 1.10)
- Nausea: 1 grade 1 probable (id 1.09)
- Vomiting: 1 grade 1 probable (Id 1.09)
